# By Regulating Mitochondrial Ca^2+^-Uptake UCP2 Modulates Intracellular Ca^2+^

**DOI:** 10.1371/journal.pone.0148359

**Published:** 2016-02-05

**Authors:** Lukas Jaroslaw Motloch, Robert Larbig, Tina Gebing, Sara Reda, Astrid Schwaiger, Johannes Leitner, Martin Wolny, Lars Eckardt, Uta C. Hoppe

**Affiliations:** 1 Department of Internal Medicine II, Paracelsus Medical University, Salzburg, Austria; 2 Division of Electrophysiology, Department of Cardiovascular Medicine, University Hospital Muenster, Muenster, Germany; University of Newcastle, AUSTRALIA

## Abstract

**Introduction:**

The possible role of UCP2 in modulating mitochondrial Ca^2+^-uptake (mCa^2+^-uptake) via the mitochondrial calcium uniporter (MCU) is highly controversial.

**Methods:**

Thus, we analyzed mCa^2+^-uptake in isolated cardiac mitochondria, MCU single-channel activity in cardiac mitoplasts, dual Ca^2+^-transients from mitochondrial ((Ca^2+^)m) and intracellular compartment ((Ca^2+^)c) in the whole-cell configuration in cardiomyocytes of wild-type (WT) and UCP2^-/-^ mice.

**Results:**

Isolated mitochondria showed a Ru360 sensitive mCa^2+^-uptake, which was significantly decreased in UCP2^-/-^ (229.4±30.8 FU vs. 146.3±23.4 FU, *P<0*.*05*). Single-channel registrations confirmed a Ru360 sensitive voltage-gated Ca^2+^-channel in mitoplasts, i.e. mCa1, showing a reduced single-channel activity in UCP2^-/-^ (Po,total: 0.34±0.05% vs. 0.07±0.01%, *P*<0.05). In UCP2^-/-^ cardiomyocytes (Ca^2+^)m was decreased (0.050±0.009 FU vs. 0.021±0.005 FU, *P<0*.*05*) while (Ca^2+^)c was unchanged (0.032±0.002 FU vs. 0.028±0.004 FU, *P>0*.*05*) and transsarcolemmal Ca^2+^-influx was inhibited suggesting a possible compensatory mechanism. Additionally, we observed an inhibitory effect of ATP on mCa^2+^-uptake in WT mitoplasts and (Ca^2+^)m of cardiomyocytes leading to an increase of (Ca^2+^)c while no ATP dependent effect was observed in UCP2^-/-^.

**Conclusion:**

Our results indicate regulatory effects of UCP2 on mCa^2+^-uptake. Furthermore, we propose, that previously described inhibitory effects on MCU by ATP may be mediated via UCP2 resulting in changes of excitation contraction coupling.

## Introduction

Mitochondrial Ca^2+^ handling is a key regulator of several important processes in cellular physiology. It has been shown to control the rate of mitochondrial energy (adenosine triphosphate, ATP) production [1, 2], modulates the spatial and temporal profile of intracellular Ca^2+^ signaling [3–5], regulates mitochondrial reactive oxygen species (ROS) generation [6] and may trigger cell death [3, 7, 8].

Mitochondrial Ca^2+-^uptake (mCa^2+^-uptake) is thought to be mostly mediated by the mitochondrial calcium uniporter (MCU) [4, 9, 10]. However, other mCa^2+^-uptake mechanisms have been suggested [11]. Electrophysiological characteristics of MCU referred to as I_MiCa_ and I_mCa1_ have been reported for COS 7 cells and diverse tissues including human myocardium, respectively [4, 10, 12]. The MCU is known to be blocked by low concentrations of Ruthenium Red (RuRed) or by its more specific derivate Ruthenium360 (Ru360) [4, 10, 13, 14]. Moreover, adenine nucleotides have been suggested to suppress MCU activity, with ATP being the most efficient inhibitor [15, 16]. However, the details of this mechanism remain unclear.

Uncoupling proteins (UCPs) are located in the inner mitochondrial membrane and belong to a superfamily of mitochondrial ion transporters [17]. UCP1 is predominately expressed in brown fat tissue where it induces a proton leak, uncouples oxidative phosphorylation and accounts for heat production [17]. UCP2 expression was identified in many tissues including the heart [13, 17, 18]. UCP2 is known to regulate ROS production (19-[19] and to modulate insulin secretion from pancreatic islets by controlling the cellular ATP concentration [20, 21]. Similar to the MCU, UCP2 is also being blocked by ATP and with a lower efficiency by other nucleotides [17, 18, 22]. However, in contrast to UCP1 the precise function of UCP2 is not yet fully understood. Recent studies indicated that UCP2 might be involved in mCa^2+^-uptake [23–25], i. e. Ca^2+^-uptake of isolated liver mitochondria from UCP2^-/-^ mice was reported to be RuRed-insensitive and enhanced mCa^2+^-uptake was observed in an endothelial cell line overexpressing UCP2. Therefore, UCP2 was postulated to be fundamental for mCa^2+^-uptake [23]. However, these results could not be replicated by others [13]. Thus, to clarify this situation we evaluated electrophysiological properties of the MCU, and investigated mitochondrial and cytosolic Ca^2+^-homeostasis from UCP2^-/-^ mice and wild-type controls.

Our data shows that the MCU may mediate Ca^2+^ flux independent of the presence of UCP2, but that these Ca^2+^-currents are modulated by UCP2 in an ATP dependent manner. Our experiments also reveal a new compensational mechanism in UCP2^-/-^ mice, which prevents possible cytosolic Ca^2+^ overload through inhibition of transsarcolemmal Ca^2+^-influx.

## Materials and Methods

### Animals

Animals were euthanized by cervical dislocation and hearts were obtained from the mouse strain B6.129S4-Ucp2^tm1Lowl^/J (UCP2^-/-^; male, n = 238) purchased from Charles River Laboratories, Research Models and Services, Germany [26]. Hearts from the identical background strain were acquired as control (WT; male, n = 195). Animals were housed in the facilities of the Paracelsus Medical University, Salzburg. The implementation of the experiments conformed to the Guide for the Care and Use of Laboratory Animals published by the US National Institutes of Health (NIH publication No. 85–23, revised 1996). The study was approved by the Ethical Committee of the Department II of Internal Medicine, Paracelsus Medical University, Salzburg according to the guidelines for the care and use of laboratory animals of the Paracelsus Medical University and the European Union.

The animals used in this study were between 8 and 12 weeks of age. To verify genetic knockout of UCP2 mice tails were genotyped using primers UCP2f2 (CAGCCACTGTGAAGTTCCTGG) and UCP2r2 (CATTGTGACACACACTTAATG) for UCP2 as well as primers GAPDH-f (AGGCCGGTGCTGAGTATGTC) and GAPDH-r (TGCCTGCTTCACCACCTTCT) for GAPDH.

### Preparation of murine cardiomyocytes

Hearts were obtained from WT or UCP2^-/-^ mice and single ventricular myocytes were isolated from murine hearts by enzymatic digestion, as previously described [27, 28]. For whole-cell experiments cardiomyocytes were stored in Dulbecco´s Modification of Eagle’s Medium (DMEM; Mediatech, Herndon, VA, USA) supplemented with 5% fetal bovine serum (Life Technologies, Carlsbad, CA, USA), 1% penicillin-streptomycin (Life Technologies, Carlsbad, CA, USA), and 15 mM HEPES, pH 7.4, in a 5% CO2 incubator at 37°C, as previously described [9]. Isolated cells were used within 6 h.

### Isolation of murine cardiac mitochondria

Cardiac mitochondria were isolated from WT or UCP2^-/-^ mice by differential centrifugation, by a modified protocol, as previously reported [29–31]. Briefly, after thoracotomy, hearts were rapidly excised into an ice-cold homogenization buffer containing: 100 mM KCl, 50 mM MOPS, 5 mM MgSO_4_ and 1 mM EGTA, pH 7.4 adjusted with KOH, atria were removed. The ventricular myocardium was minced in a KB buffer on ice containing: 143 mM KCl, 70 mM glucose, 20 mM taurine, 5 mM HEPES, 0.5 mM EGT, BSA 0.1%, pH 7.2 adjusted with KOH and incubated with 1500 U/ml collagenase B (Roche, Basel, Switzerland) at 37°C for 30 minutes. Subsequently the homogenate was centrifuged for 10 min at 750 g. The supernatant, containing the mitochondrial fraction, was further centrifuged at 7.000 g for 10 min, and the pellet was suspended in 1 ml of standard incubation buffer containing: 125 mM KCl, 20 mM HEPES, 15 mM NaCl_2_, 5 mM Na-succinate, 5 mM MgCl_2_, 2 mM K_2_HPO_4_, pH 7.0 adjusted with KOH and spun at 7.000 g for 10 min. Mitochondria were resuspended in standard incubation buffer. Protein concentration was determined using BCA Assays (Thermo Scientific, Rockford, IL, USA). Protein concentration was adjusted to 1 mg/ml. Mitochondria were kept on ice before experiments.

### Preparation of mitoplasts

Isolated intact cardiac subsarcolemmal mitoplasts were prepared from isolated myocytes by differential centrifugation, as previously reported [4, 10, 32]. Freshly isolated cardiomyocytes were used within 1–2 h. Myocytes were labeled with Mitotracker Green 1 μM (Life Technologies, Carlsbad, CA, USA) to facilitate identification of intact mitoplasts after further subcellular purification [4, 10, 32]. Mitochondria were stored at 4°C for up to 24 h for patch-clamp experiments. Mitoplasts were prepared from intact mitochondria prior to patching or protein preparation by osmotic shock, as previously described [4, 10, 32].

### Respiratory control rate

Isolated mitochondria were suspended in the standard incubation buffer (125 mM KCl, 2 mM K_2_HPO_4_, 20 mM HEPES, 5 mM Na-succinate, 5 mM MgCl_2_, pH 7.0 adjusted with KOH) and then transferred to a water-jacketed and magnetically stirred oxygraph chamber of an Oroboros Oxygraph-2k (Oroboros Instruments GmbH, Innsbruck, Austria). Mitochondrial quality was assessed by measuring the respiratory control rate (RCR), using 1 mM ADP (state 3) or 1 mM ADP and 2.5 mg/ml oligomycin (pseudo state 4). Mitochondria consistently had RCR values between 4 and 7 with complex I substrates. The data were analyzed, as previously described [33].

### Measurements of mitochondrial membrane potential

Experiments were performed as previously described using a modified protocol [34–38]. Mitochondrial membrane potential was estimated by measuring transmembrane distribution of tetramethyl-rhodamine methyl ester (TMRM). Briefly, isolated mitochondria were suspended in a potassium buffer (140 mM KCl, 100 mM HEPES, 5 mM K_2_HPO_4_, 1.5 mM malate, 1.5 mM pyruvate, 1.5 mM glutamate, pH with 7.4 adjusted with KOH) and incubated with 2 nM TMRM for 30 min to achieve transmembrane equilibrium of the dye [34, 37]. Mitochondria were placed in a heated (37°C) Tecan Infinte200 PRO plate reader (Tecan Group Ltd., Männedorf, Switzerland). TMRM fluorescence was measured for 60 s. To validate the approach CCCP (500 nM; carbonyl cyanide m-chlorophenyl hydrazone) was added to depolarize mitochondria, mitochondria were incubated for 2 min and the fluorescence was recorded for 30 s. TMRM was excited at λ_exc_ = 574 nm and emission was measured at λ_em_ = 590 nm. Only experiments which revealed a significant decrease in fluorescence signal after CCCP application indicating depolarization of mitochondria were included.

### Measurements of mCa^2+^-uptake

Isolated mitochondria were suspended in standard incubation buffer and loaded with rhod-2-acetoxymethyl ester (rhod2-AM; Life Technologies, Carlsbad, CA, USA) for 30 minutes at 4°C to determine mCa^2+^-uptake, as previously described [39]. 1 μM rotenone and 3 μM oligomycin were added to maximize mitochondrial membrane potential. Specific drugs were added to the test solution to block the mitochondrial permeability transition pore (10 μM cyclosporine A) and the mitochondrial NCX (10 μM CGP-37157). Finally, mitochondria were placed in a heated (37°C) Tecan Infinte200 PRO plate reader (Tecan Group Ltd., Männedorf, Switzerland). In each experiment 200 nM Ca^2+^ were added using an automated infusion system and rhod2-AM fluorescence was measured. Rhod2-AM was excited at λ_exc_ = 540 nm and emission was measured at λ_em_ = 605 nm. Data was acquired for 660 s.

### Single-channel recordings

All experiments were performed in the mitoplast-attached configuration of the patch-clamp technique (at least 60 test pulses of 150-ms duration at 1.67 Hz, if not indicated otherwise; sampling frequency, 10 kHz; corner frequency, 2 kHz). The bath solution contained 160 mM KCl, 10 mM HEPES, 1 mM EDTA, 1 mM EGTA, pH 7.2 adjusted with KOH. Pipettes were filled with a solution containing 105 mM CaCl_2_, 10 mM HEPES, pH 7.2 adjusted with Ca(OH)_2_ [4, 10, 32]. Specific drugs were added to solutions to block the mitochondrial permeability transition pore (10 μM cyclosporine A, Sigma Aldrich, St. Louis, MO, USA), the mitochondrial ryanodine receptor (RyR; 10 μM dantrolene, Sigma Aldrich, St. Louis, MO, USA), the inositol triphophate receptor (IP_3_R; 10 μM xestospongin C, Sigma Aldrich, St. Louis, MO, USA), the mitochondrial Na^+^-Ca^2+^ exchanger (NCX; 10 μM CGP-37157, Calbiochem, San Diego, CA, USA) [4, 10, 32]. Ru360 (Merck, Darmstadt, Germany) and ATP (1 mM, Sigma Aldrich, St. Louis, MO, USA) were used as indicated. Currents were recorded and digitized with an Axopatch 200B amplifier and Digidata 1200 interface (MDS Analytical Technologies, Toronto, Canada), as previously described [27, 40].

### Single-channel analysis

Single-channel analysis was done using custom software only from single-channel patches, as previously reported [10, 32]. Briefly, linear leak and capacity currents were digitally subtracted using the average currents of non-active sweeps. For detailed gating analysis idealized currents were analyzed in 150 ms steps. Active sweeps were defined as those with at least one opening. The total open probability (Po,total; defined as the occupancy of the open state during the total pulse duration) was analyzed for at least 3 s pulse durations at -100 mV of 60 sweeps with 150 ms duration. Single-channel amplitudes were determined by direct measurements of fully resolved openings for conductance calculations or as the maximum of Gaussian fits to amplitude histograms

### Whole-cell patch-clamp experiments

#### Determination of (Ca^2+^)c and (Ca^2+^)m

Ca^2+^ measurements were performed, as previously described [9]. Briefly, isolated murine cardiomyocytes were incubated with 3 μM rhod2-AM to monitor (Ca^2+^)m. Using the patch-clamp technique, cytosolic traces of rhod2-AM were eliminated by whole-cell dialysis with a pipette solution containing 50 μM fluo4-pentapotassium-salt (fluo4-K5, Life Technologies, Carlsbad, CA, USA) to monitor (Ca^2+^)c [6, 9, 41]. The pipette solution contained 130 mM K-glutamate, 19 mM KCl, 0.5 mM MgCl_2_, 5 mM Na-HEPES, 10 mM HEPES, 5 mM ATP, and 0.05 mM of the cell-impermeable fluo4-K5. Myocytes were equilibrated for >10 min. Thus, (Ca^2+^)m was reported by rhod2-AM and (Ca^2+^)c by fluo4-K5 [6, 9]. Where indicated, the pipette solution contained 10 nM Ru 360 or 100 nM Ru360 [6, 9, 10, 14], or 5 mM ATP (control), 15 mM ATP, or was an ATP-free solution. Myocytes were placed on cover slips that were mounted in a heated recording chamber (37°C) on the stage of a fluorescence microscope (Zeiss Axiovert 200, Carl Zeiss Microscopy GmbH, Jena, Germany) and superfused with a modified Tyrode´s solution containing: 130 mM NaCl, 5 mM KCl, 1 mM MgCl_2_, 10 mM Na-HEPES, 2 mM CaCl_2_, 10 mM glucose, 2 mM pyruvate, 0.3 mM ascorbic acid, pH 7.4 adjusted with NaOH. All myocytes were whole-cell voltage-clamped and Ca^2+^ transients were elicited by a rectangular pulse from -80 mV to 10 mV for 300 ms at 0.5 Hz, as described previously [42]. Membrane currents were recorded, as previously described [42] in whole-cell voltage-clamp mode (Axopatch 200B amplifier, Digidata 1200B interface, MDS Analytical Technologies, Toronto, Canada) with 2–4 MΩ pipettes. Electrophysiological signals were acquired, stored and analyzed using pClamp 9 software (MDS Analytical Technologies, Toronto, Canada), as previously described [9, 43]. Series resistance compensation was applied to all recordings. Cardiomyocytes were stimulated at 1 Hz for 2 min using rectangular pulses from -80 mV to 10 mV prior to the recording of Ca^2+^ transients to eliminate cytosolic traces of rhod2-AM and normalize sarcoplasmatic reticulum (SR) Ca^2+^ load, as previously described [9]. Between each conditioning pulse, membrane potential was held at -80 mV.

To measure (Ca^2+^)c, fluo4-K5 was excited at λ_exc_ = 470 nm, and emission was collected at λ_em_ = 520 nm, as described previously [41], with a custom built, two channel photomultiplier tube and an xenon arc lamp light source (Till Photonics, Graefeling, Germany). To determine (Ca^2+^)m, rhod2-AM was excited at λ_exc_ = 550 nm, and fluorescence (F) was recorded at λ_em_ = 605 nm.. Fluorescence data was normalized to F0 after completion of the stimulation protocol described above (F/F0). When necessary, (Ca^2+^)m and (Ca^2+^)c signals were corrected for rhod2-AM bleaching (~2% per minute) and fluo4-K5 bleaching (~10% per minute), respectively.

#### Determination of transsarcolemmal Ca^2+^-influx

To measure transsarcolemmal Ca^2+^-influx we used a modified protocol, as previously described [43]. Briefly, to measure transsarcolemmal Ca^2+^-influx we isolated cardiomyocytes and established the whole cell configuration using the patch clamp technique. For these experiments a modified pipette solution was used containing 0.2 μM ryanodine and 0.01 mM thapsigargin, as previously described to block RyR and the SR Ca^2+^-ATPase (SERCA) [43]. Additionally, 100 nM Ru360 was used in the pipette solution to avoid possible modulations of dyadic cleft Ca^2+^ by mCa^2+^-uptake. By using the stimulation protocol, fluo4-K5 and the detectors as described above we recorded (Ca^2+^)c. When necessary traces were filtered using a 30–50 Hz low pass Gaussian filter.

#### Determination of I_NCX_ and SR Ca^2+^ load

SR Ca^2+^ load was registered as previously described [44]. Briefly, we used a bath solution containing 136 mM NaCl, 5.4 mM KCl, 10 mM HEPES, 1 mM MgCl_2_, 0.33 mM NaH_2_PO_4_, 1 mM CaCl_2_, 10m M glucose, pH 7.4 adjusted with NaOH and an internal solution containing 110 mM CsCl, 30 mM TEA-Cl, 10 mM NaCl, 10 mM HEPES, 5 mM MgATP, pH 7 adjusted with CsOH. Miniature solenoid valves (NPI Electronic GmbH, Hamm, Germany) controlled by PCLAMP digital outputs controlled the rapid solution exchange with an additional 5 mM caffeine in the bath solution. This enabled precise timing of solution exchanges in relation to the voltage clamp protocol. To quantify SR Ca^2+^ load in WT and UCP2^-/-^ myocytes, we recorded I_NCX_ during an application of 5 mM caffeine for 1 s. We administered a train of five prepulses from -40 to 0 mV at 1 Hz lasting 100 ms immediately before caffeine application to ensure a steady-state SR Ca^2+^ load. A constant holding potential of -40 mV was maintained during exposure to caffeine. SR Ca^2+^ load was analyzed by calculating the integral of the I_NCX_ current recordings.

### Statistical analysis

In experiments obtained in isolated mitochondria n refers to the number of animals. In whole and single patch clamp experiments n refers to the number of patch-experiments obtained in mitoplasts or cardiomyocytes isolated from a minimum of three hearts. Pooled data are presented as mean ± SEM. Comparisons between two groups were performed with unpaired t-test and multiple comparisons with one-way ANOVA followed by Bonferroni test. Probability values of *P*<0.05 were regarded significant.

## Results

### Reduced Ca^2+^-uptake in isolated cardiac mitochondria of UCP2^-/-^ vs. WT mice

**Fig 1:**
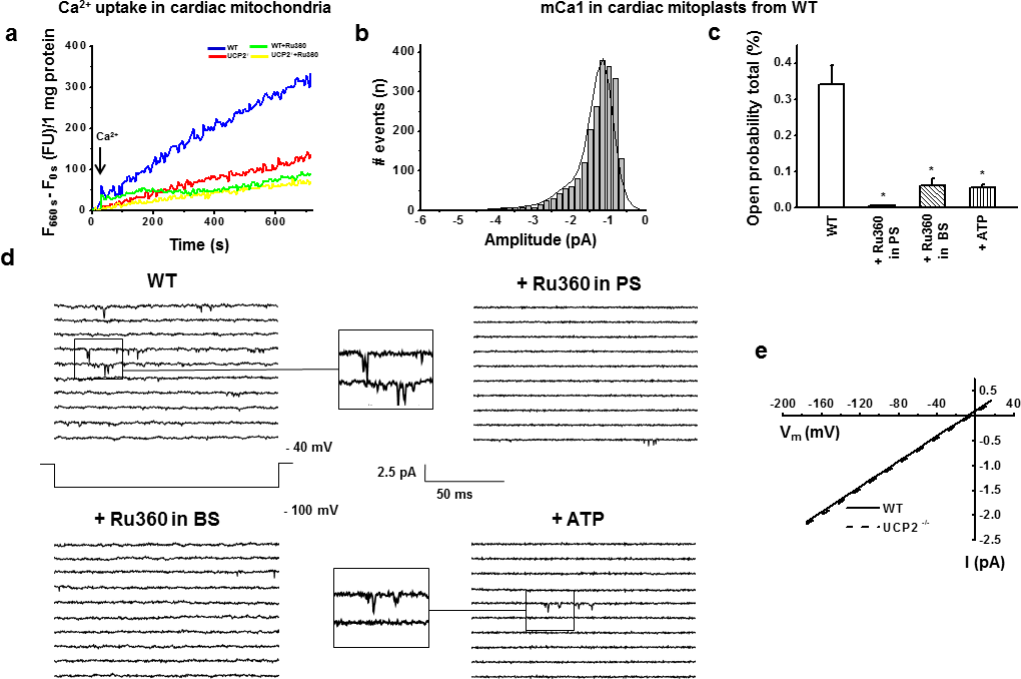
a) Original traces of recordings of Ca^2+^-uptake in rhod2-AM loaded isolated cardiac mitochondria from WT and UCP2^-/-^ after a bolus of 200 nM Ca^2^. b) Amplitude histogram of basic mCa1 in mitoplasts from WT. Basic mCa1 in WT showed three amplitude levels with -1.16±0.02 pA being the most common observed amplitude [I_unitary_: -1.16±0.02 pA, n = 30; μ_1_: -1.16 pA (65%), μ_2_: -1.71 pA (31%), μ_3_: -3.10 pA (4%)]. c) In cardiac mitoplasts from WT Ru360 (200 nM) in PS (n = 8) and Ru360 (10μM) in BS (n = 5) as well as ATP (1mM) in BS (n = 5) decreased total open probability (Po,total) of mCa1 (*p<0.05). d) Examples of consecutive original traces of cardiac mCa1 in mitoplasts from WT: mCa1 in WT vs. mCa1 in WT + Ru360 (200 nM) in pipette solution (PS) vs. mCa1 in WT + Ru360 (10 μM) in bath solution (BS) vs. mCa1 in WT + ATP (1mM) in BS. e) Slope conductance of mCa1 in WT (12.59±1.57 pS, n = 17) and of mCa1 in UCP2^-/-^ (12.79±1.83 pS, n = 13) was not different.

To evaluate the role of UCP2 for Ca^2+^-uptake in intact mitochondria we measured rhod2-AM fluorescence upon Ca^2+^ application (200 nM). Mitochondria had respiratory control rates from 4 to 7 with complex I substrates [33]. WT and UCP2^-/-^ mitochondria revealed no difference in TMRM load (F_TMRM_: 31260.2±2318.1 FU, n = 11, WT vs. F_TMRM_: 31712.6±2320.9 FU, n = 9, UCP2^-/-^, *P*>0.05) indicating a similar membrane potential in both groups. To rule out unspecific effects of other known mCa^2+^uptake mechanisms, mitochondria were incubated with various mitochondrial Ca^2+^-channel blockers (see methods section for details). Both WT and UCP2^-/-^ mitochondria showed a Ru360 (100 nM) sensitive Ca^2+^-uptake (F_WT+Ru360_/F_WT_: 45.1%, n = 9, *P*<0.05; F_UCP2_^-/-^_+Ru360_/F_UCP2_^-/-^: 56.2%, n = 7, *P*<0.05; Fig 1A). However, compared to WT mitochondria UCP2^-/-^ mitochondria revealed a significantly lower Ca^2+^-influx (F_660s_- F_0s_: 229.4±30.8 FU, n = 9, WT vs. 146.3±23.4 FU, n = 7, UCP2^-/-^ *P*<0.05; Fig 1A).

### Characterization of mCa1 single channel activity in subsarcolemmal, cardiac mitoplasts of WT mice ± Ru360 and ATP

By patch-clamping the inner membrane of subsarcolemmal mitoplasts prepared from isolated cardiomyocytes, in 26% of total patches we verified the existence of murine mitochondrial mCa1 channels in WT mice. We detected voltage-dependent single-channel currents with a unitary conductance of 12.59±1.57 pS, three different amplitude sublevels with -1.16±0.02 pA being the most common, a total open probability of 0.34±0.05% (Po,total; at -100 mV, n = 30) (Fig 1B–1E, Table 1). After continuous hyperpolarization to -100 mV the channel did not exhibit time-dependent inactivation or run-down over 48 s of hyperpolarization (Po,total in time period 0–12 s/ Po,total in time period 36–48 sec: 95.0±7.1%, n = 3). Thus, during all patch-clamp experiments mitoplasts were hyperpolarized within this period of time. Ru360 significantly decreased the total open probability (Po,total: 0.03±0.01%, n = 8, *P*<0.05 using 200 nM Ru360 in the pipette solution and Po,total: 0.06±0.02%, n = 5, *P*<0.05 using 10 μM Ru360 in the bath solution) without affecting single-channel amplitude (Table 1). Thus, murine mCa1 could be inhibited by Ru360 in concentrations, which are known to block specifically the MCU [4, 7, 10, 45, 46]. Moreover, mCa1 channel activity was also inhibited by 1 mM ATP in the bath solution which is known to decrease the MCU activity [7, 15, 16]. ATP suppressed mCa1 channel activity by reducing the open probability, total (Po,total; 0.06±0.01%, n = 5, *P*<0.05) (Fig 1C–1D, Table 1).

**Table 1:** Gating parameters of mCa1 in WT.

	WT Control	WT+Ru360 (200 nM) in PS	WT+Ru360 (10 μM) in BS	WT+ATP (1 mM) in BS
Open probability, total [%]	0.341±0.053	0.029±0.008 *	0.065±0.015 *	0.055±0.010 *
Mean open time [ms]	0.313±0.016	0.248±0.018	0.242±0.007	0.262±0.015
Mean first latency [ms]	55.779±1.874	66.146±5.011	64.618±2.126	59.388±3.555
Amplitude/ I_unitary_ [pA]	-1.163±0.023	-1.123±0.047	-1.170±0.034	-1.130±0.040
No. Experiments	30	8	5	5

Gating parameters of mCa1 channels and effects of Ruthenium 360 (Ru360) in pipette solution (PS; 200 nM), Ru360 in bath solution (BS; 10 μM) and ATP (1 mM) in bath solution on mCa1 single-channel characteristics in WT mitoplasts. Holding potential -40 mV, test potential -100 mV.

* p<0.05 vs. WT.

### Characterization of reduced mCa1 single channel activity in subsarcolemmal cardiac mitoplasts of UCP2^-/-^ mice ± Ru360 and ATP

**Fig 2:**
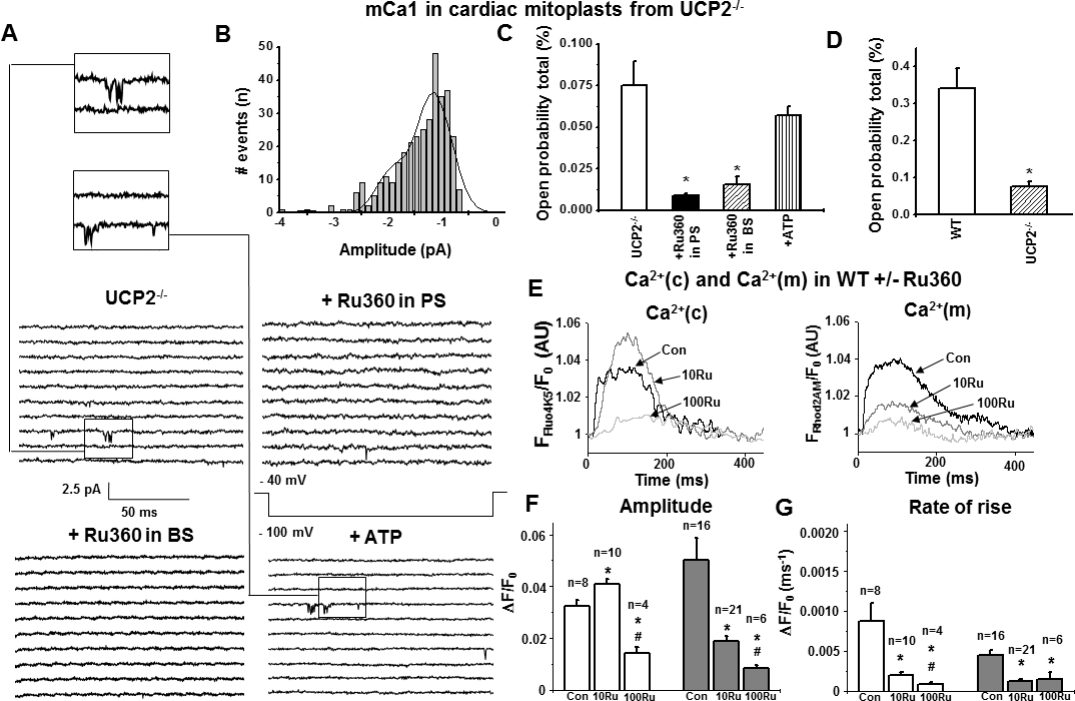
a) Examples of consecutive original traces of cardiac mCa1 in mitoplasts from UCP2^-/-^: mCa1 in UCP2^-/-^ vs. mCa1 in UCP2^-/-^ + Ru360 (200 nM) in PS vs. mCa1 in UCP2^-/-^ + Ru360 (10 μM) in BS vs. mCa1 in UCP2^-/-^ + ATP (1 mM) in BS. b) Amplitude histogram of basic mCa1 in cardiac UCP2^-/-^ mitoplasts. Basic mCa1 in UCP2^-/-^ showed three amplitude levels with -1.19±0.04 pA being the most common observed amplitude [I_unitary_: -1.19±0.04 pA, n = 23; μ_1_: -1.16 pA (78%), μ_2_: -1.95 pA (21%), μ_3_: -3.51 pA (1%)]. c) In cardiac mitoplasts from UCP2^-/-^ Ru360 (200 nM) in pipette solution (PS, n = 7) and Ru360 (10 μM) in bath solution (BS, n = 4) significantly decreased total open probability (Po, total) of mCa1 (*p<0.05). ATP (1 mM, n = 7) had no effect on mCa1 activity in UCP2^-/-^. d) Total open probabilities (Po, total) of basic mCa1: In comparison to WT mCa1, Po,total of mCa1 in UCP2^-/-^ was significantly decreased (*p<0.05). e-g) Ca^2+^ transients in WT cardiomyocytes (Con) ± 10 and 100 nM Ru360. e) Representative traces of (Ca^2+^)c (left) and (Ca^2+^)m (right), f-g) Statistical analysis: In WT cardiomyocytes 10 nM Ru360 (10Ru) significantly elevated amplitude while (Ca^2+^)c rate of rise was decreased. 100 nM Ru360 (100Ru) suppressed both parameters. In mitochondria amplitude and rate of rise of (Ca^2+^)m were significantly decreased using either 10 or 100 nM Ru360. *p<0.05 vs. Con; ^#^p<0.05 vs. Con + 10 nM Ru360.

To clarify the role of UCP2 in mCa^2+^-uptake and its potential regulative influence on mitochondrial Ca^2+^-uptake, we analyzed single-channel currents in cardiac mitoplasts of UCP2^-/-^ mice. Notably, we recorded a Ca^2+^-current with a similar unitary conductance compared to mCa1 channels of WT mitoplasts (12.79±1.83 pS; *P*>0.05. vs. WT). mCa1 occurred in 38% of total patches and compared to WT showed a trend towards an increase in the probability of occurrence (ratio of active to total patches) in the UCP2^-/-^ group, however, without reaching statistical significance (*P = 0*.*08*, calculated by Chi-square test). The channel exhibited three different amplitude sublevels with -1.19±0.04 pA being the most common (*P*>0.05 vs. WT). However, the total open probability was significantly lower (Po,total: 0.07±0.01%, n = 23, *P*<0.05) in UCP2^-/-^ mitoplasts compared to mCa1 obtained from WT mitoplasts, indicating mCa1 to be present but less active in UCP2^-/-^ mitochondria (Figs 1E and 2A–2D, Table 2). Consistent with murine WT, mCa1 in UCP2^-/-^ mice was also inhibited by 200 nM Ru360 in the pipette solution and by 10 μM Ru360 in the bath solution (Fig 2A and 2C, Table 2).

**Table 2:** Gating parameters of mCa1 in UCP2^-/-^.

	UCP2^-^/^-^	UCP2^-^/^-^+Ru360 (200 nM) in PS	UCP2^-^/^-^+Ru360 (10 μM) in BS	UCP2^-^/^-^+ATP (1 mM) in BS
Open probability, total [%]	0.075±0.015	0.009±0.001 *	0.016±0.005 *	0.057±0.006
Mean open time [ms]	0.290±0.021	0.209±0.034	0.175±0.018	0.346±0.048
Mean first latency [ms]	62.642±2.510	69.640±6.730	76.695±6.276	58.703±4.119
Amplitude/ I_unitary_ [pA]	-1.194±0.042	-1.176±0.080	-1.338±0.088	-1.160±0.045
No. Experiments	23	7	4	7

Gating parameters of mCa1 channels and effects of Ruthenium 360 (Ru360) in pipette solution (PS; 200 nM), Ru360 in bath solution (BS; 10 μM) and ATP (1 mM) in BS on mCa1 single-channel characteristics in UCP2^-/-^ mitoplasts. Holding potential -40 mV, test potential -100 mV.

* p<0.05 vs. UCP2^-/-^ control.

The observation that UCP2 may regulate mCa1 activation and the fact that ATP is known to inhibit both UCP2 and mCa^2+^-uptake [15–18, 22], led us to the conclusion that inhibition of mCa1 by ATP might be mediated via UCP2. Thus, we evaluated mCa1 responsiveness to ATP concentrations known to inhibit UCP2 (1 mM ATP in bath solution). In contrast to WT, mCa1 single-channel activity of mCa1 in UCP2^-/-^ mitoplasts was completely insensitive to ATP (Po,total, 0.06±0.01%, n = 7, *P*>0.05, Fig 2A and 2C, Table 2).

### Concentration-dependent effects of Ru360 on mitochondrial and cytosolic Ca^2+^ transients in isolated cardiac myocytes from WT mice

In whole-cell patch-clamped cardiomyocytes from WT mice we measured fluo4-pentapotassium-salt (fluo4-K5) mediated cytosolic and rhod2-AM mediated mitochondrial Ca^2+^ transients elicited by membrane depolarization from -80 mV to 10 mV. To verify our findings from single-channel analysis and isolated mitochondria we exposed WT cardiomyocytes to 10 and 100 nM Ru360 via the pipette solution, respectively. Consistent with a previous report [9] using very low concentrations of Ru360 (10 nM), we observed a decrease in the amplitude of mitochondrial Ca^2+^ transients and a significant increase of the amplitude in cytosolic Ca^2+^ transients (Fig 2E–2G). Furthermore, the rate of rise of mitochondrial Ca^2+^ transients was significantly reduced in the presence of 10 nM Ru360 in mitochondria and the cytosolic compartment (Fig 2E and 2G). Ru360 in concentrations of 100 nM, significantly decreased both the amplitude and rate of rise of mitochondrial and cytosolic Ca^2+^ transients vs. control (Fig 2E–2G). Inhibition of mCa^2+^-uptake therefore has a profound regulatory effect on cytosolic Ca^2+^ transients and thus, presumably on cardiac contractility under steady state conditions.

### Decreased mitochondrial and unchanged amplitude of cytosolic Ca^2+^ transients in isolated cardiac myocytes of WT vs. UCP2^-/-^

**Fig 3:**
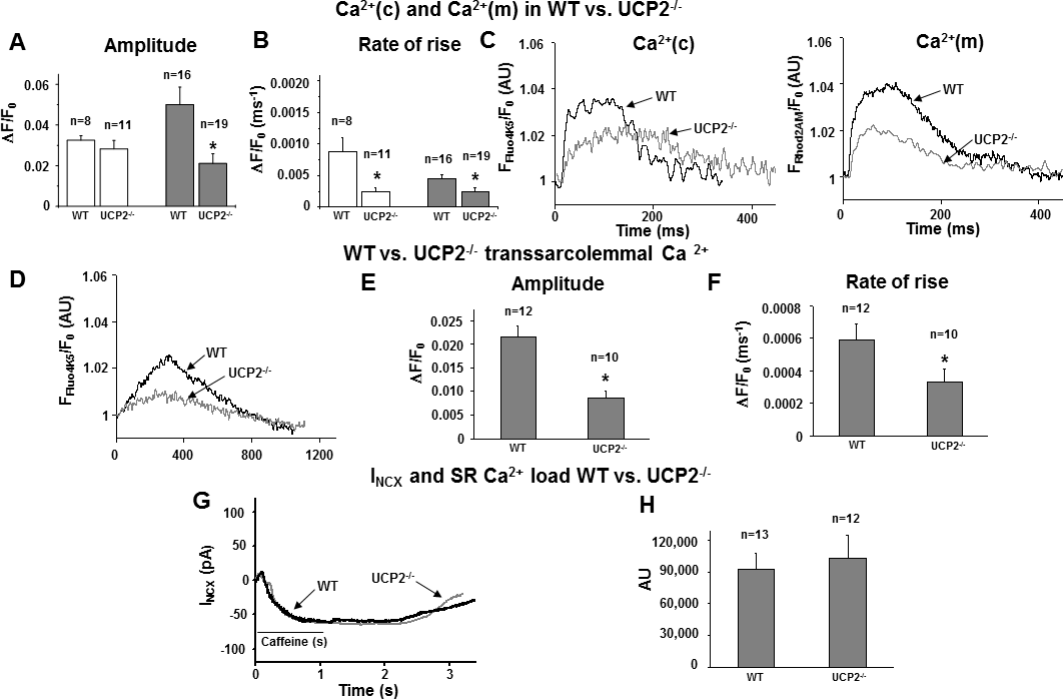
a-c) Ca^2+^ transients in wild-type (WT) and in UCP2^-/-^ cardiomyocytes (UCP2). a-b) Statistical analysis of (Ca^2+^)c (left) and (Ca^2+^)m (right) and c) representative traces of (Ca^2+^)c (left) and (Ca^2+^)m (right): In UCP2^-/-^ cardiomyocytes rate of rise of (Ca^2+^)c was significantly decreased while the amplitude of (Ca^2+^)c was unchanged vs. WT (*p<0.05). The amplitude and rate of rise of (Ca^2+^)m of UCP2^-/-^ cardiomyocytes were significantly decreased vs. WT (*p<0.05). d-f) Transsarcolemmal Ca^2+^ transients in WT vs. UCP2^-/-^ cardiomyocytes evoked in the presence of 0.2 μM ryanodine, 0.01 mM thapsigargin and 100 nM Ru360. d) Representative traces of transsarcolemmal (Ca^2+^)c in WT and UCP2^-/-^, and e-f) Statistical analysis of amplitude (e) and rate of rise (f): In UCP2^-/-^ transsarcolemmal Ca^2+^ transient amplitude and rate of rise were significantly down-regulated (*p<0.05 vs. WT). g) I_NCX_ WT vs. UCP2^-/-^ cardiomyocytes: forward I_NCX_ was elicited by 1 s of exposure to 5 mM caffeine using a holding potential of -40 mV. No difference in caffeine induced I_NCX_ in WT compared to UCP2^-/-^ was found. h) SR Ca^2+^ load in WT vs. UCP2^-/-^ calculated via the integral of I_NCX_ recordings. No difference in SR Ca^2+^ load between WT and UCP2^-/-^ was detected.

To assess the impact of UCP2-depletion on mCa^2+^-uptake and EC coupling under physiological conditions we recorded dual Ca^2+^ transients from intact UCP2^-/-^ cardiomyocytes. Consistent with the single-channel data UCP2^-/-^ cardiomyocytes showed a significantly lower amplitude and slower rate of rise of mitochondrial Ca^2+^ transients vs. WT. The amplitude of (Ca^2+^)c was unchanged in UCP2^-/-^ vs. WT while rate of rise was reduced suggesting a counter-regulatory mechanism against cytosolic Ca^2+^ overload in UCP2^-/-^ myocytes (Fig 3A–3C).

### Reduced transsarcolemmal Ca^2+^ influx and unchanged SR loading in isolated cardiac myocytes of WT vs. UCP2^-/-^

Thus, to analyze possible compensational regulations in UCP2^-/-^ cardiomyocytes we decided to further focus on intracellular Ca^2+^-handling. First we measured systolic transsarcolemmal Ca^2+^ influx while blocking the ryanodine receptor (RyR) and SR Ca^2+^-ATPase (SERCA) using 0.2 μM ryanodine and 0.01 mM thapsigargin, respectively, as previously described [43]. Additionally, 100 nM Ru360 were present in the pipette solution to avoid possible modulations of cleft Ca^2+^ by mCa^2+^uptake via the MCU. UCP2^-/-^ cardiomyocytes presented a significantly down-regulated transsarcolemmal Ca^2+^-influx with a significantly diminished slope factor in comparison to WT (Fig 3D–3F), which thus might prevent cytosolic Ca^2+^ overload in these cells. To further evaluate this issue we examined SR Ca^2+^ load using the whole-cell patch-clamp technique in isolated cardiomyocytes. In accordance with the data obtained by measuring transsarcolemmal Ca^2+^-influx, SR Ca^2+^ load was unchanged compared to WT indicating a compensational mechanism with decreased Ca^2+^ entry to avoid intracellular Ca^2+^ overload in UCP2^-/-^ mice. (Fig 3G–3H)

### Concentration-dependent effects of Ru360 on mitochondrial and cytosolic Ca^2+^ transients in isolated cardiac myocytes from UCP2^-/-^ mice

**Fig 4:**
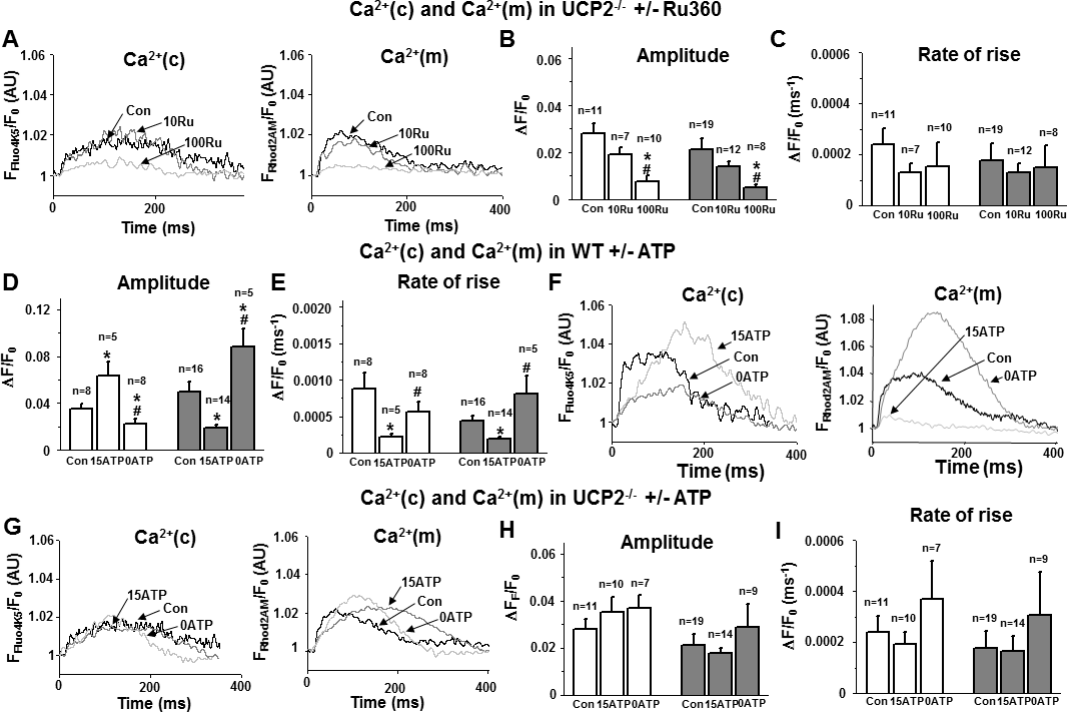
a-c) Ca^2+^ transients in UCP2^-/-^ cardiomyocytes (UCP2) ± 10 and 100 nM Ru360. a) Representative traces of (Ca^2+^)c (left) and (Ca^2+^)m (right), and b-c) Statistical analysis: 10 nM Ru360 (10Ru) had no impact on either (Ca^2+^)c or (Ca^2+^)m transient amplitudes of UCP2^-/-^ cardiomyocytes. 100 nM Ru360 (100Ru) significantly decreased the amplitude in both compartments without affecting the rate of rise. *p<0.05 vs. Con; ^#^p<0.05 vs. Con + 10 nM Ru360. d-f) Ca^2+^ transients in WT cardiomyocytes + 5 (Con), + 0 (0ATP) and + 15 mM ATP (15ATP). d-e) Statistical analysis, and f) representative traces of (Ca^2+^)c (left) and (Ca^2+^)m (right): In the absence of ATP the amplitude of (Ca^2+^)m was significantly increased, while it was suppressed when ATP was elevated to 15 mM. Conversely, the amplitude of (Ca^2+^)c was significantly decreased in the absence of ATP and significantly increased with 15 mM ATP. The rate of rise of (Ca^2+^)m and (Ca^2+^)c was significantly decreased in the presence of 15 mM ATP. This inhibitory effect of ATP suggests a similar shift of dyadic cleft Ca^2+^ towards the ryanodine receptor (RyR) as in WT cardiomyocytes ± Ru360. The opposite effect was observed in the absence of ATP. *p<0.05 vs. Con; ^#^p<0.05 vs. 15 mM ATP. g-i) Ca^2+^ transients in UCP2^-/-^ cardiomyocytes + 5 (Con), + 0 (0ATP) and + 15 mM ATP (15ATP). g) Representative traces of (Ca^2+^)c (left) and (Ca^2+^)m (right), and h-i) statistical analysis: The modulation of the ATP concentration had no significant impact on either amplitude or rate of rise of Ca^2+^ in both cellular compartments.

To further support the existence of MCU in the cardiac UCP2^-/-^ model, UCP2^-/-^ cardiomyocytes were exposed to 10 and 100 nM Ru360 via the internal solution while monitoring mitochondrial and cytosolic Ca^2+^-uptake. Interestingly, 10 nM Ru360 had no significant impact on either mitochondrial (Fig 4A–4C) or cytosolic Ca^2+^ transients (Fig 4A–4C). However, Ru360 concentrations of 100 nM almost completely abolished Ca^2+^ transients in the mitochondria and cytosol (Fig 4A–4C). We detected no significant impact on the slope factors in both compartments at Ru360 concentrations of 100 nM (Fig 4A–4C).

### ATP modulates mitochondrial and cytosolic Ca^2+^ transients in isolated cardiac myocytes from WT mice

To investigate the effect of ATP on mitochondrial and cytosolic Ca^2+^-uptake, WT cardiomyocytes were patch-clamped using either an ATP enriched (ATP 15 mM) or ATP depleted pipette solution. Mitochondrial Ca^2+^ transient amplitude and rate of rise were significantly decreased by additional delivery of high amounts of ATP (15 mM) via the pipette solution (Fig 4D–4F). Conversely, using high amount of ATP the amplitude of the cytosolic Ca^2+^ transient was increased with a significantly reduced rate of rise (Fig 4D–4F). This response was similar to the data obtained in WT cardiomyocytes exposed to 10 nM Ru360. An opposite effect with significant increase of mCa^2+^-uptake and decreased cytosolic Ca^2+^ transient (Fig 4D–4F) was observed with an ATP-free pipette solution. The rate of rise of the Ca^2+^ transients in both cellular compartments was not significantly altered vs. control using an ATP free solution (Fig 4D–4F).

### No ATP modulation of Ca^2+^ transients in isolated cardiac myocytes from UCP2^-/-^ mice

To further support our data of single channel recordings which demonstrated an inhibitory effect of ATP on the MCU, via UCP2, cardiomyocytes from UCP2^-/-^ mice were patch-clamped with ATP enriched (ATP 15 mM) and ATP free solution. Consistently neither mitochondrial Ca^2+^ transients nor cytosolic Ca^2+^ transients were significantly altered by either ATP concentration in intact cardiomyocytes from UCP2^-/-^ mice (Fig 4G–4I).

## Discussion

Since the role of UCP2 in mitochondrial Ca^2+^ handling remains inconclusive [13, 23] we aimed to evaluate whether UCP2 modulates mitochondrial Ca^2+^-uptake.

To define the role of UCP2 we used three complementary approaches in WT and UCP2^-/-^ mice: 1.) measurement of Ca^2+^-uptake in intact isolated mitochondria by using rhod-2 AM, 2.) single-channel recordings of subsarcolemmal mitoplasts prepared from isolated cardiomyocytes, and 3.) analysis of dual Ca^2+^ transients elicited by voltage-clamp depolarizations from the mitochondrial ((Ca^2+^)m) and intracellular compartment ((Ca^2+^)c) in whole-cell patch-clamped, isolated ventricular cardiomyocytes.

The experiments performed in isolated mitochondria demonstrated a Ru360 sensitive Ca^2+^-uptake in both WT and UCP2^-/-^ mitochondria. However, compared to WT mitochondria UCP2^-/-^ mitochondria showed a significantly lower Ca^2+^-uptake.

Using single-channel recordings in WT and UCP2^-/-^ mitoplasts, we detected a murine cardiac Ca^2+^-channel which presented burst like open states, a mean amplitude of -1.1–1.2 pA at -100 mV and a conductance of 12–13 pS. These characteristics are not equal to the I_MiCa_ which was proven to underlie the MCU [4, 47] and which presented a conductance of 2.6–5.2 pS and long lived open states at -160 mV in COS-7 cells [4]. However, I_MiCa_ activity was reported to differ between various tissues and species showing the lowest activity in the heart [12]. In accordance with this observation, in the human heart Michels and colleagues reported a Ru360 sensitive mCa1 channel with burst like open states, an amplitude of -1.4 pA at -100 mV and a conductance of 13–14 pS [10]. A channel with analog single channel properties (burst open states, amplitude around -1 pA at -100 mV and a conductance of 11–12 pS) was also described by the Graier laboratory which characterized three mitochondrial Ca^2+^-channels in mitoplasts isolated from HeLa cells (*i*-MCC, *xl*-MCC and *b*-MCC) [48]. The authors concluded, that the most frequently observed *i*-MCC matches the human cardiac mCa1 [48]. Since, its activity was shown to be dependent on siRNA-mediated diminution of MCU and the channel was described as ruthenium sensitive, they reasoned, that this type of activity is indicative for the MCU-established current [49]. Thus, we suggest that in this work we characterized the murine cardiac mCa1 which corresponds to the human cardiac mCa1 and to the *i*-MCC described in HeLa cells. Therefore, this channel might display activity characteristics probably suggestive for the MCU-established current in the murine heart [10, 49].

Consistent with intact mitochondria we found a mCa1 current with a high sensitivity to Ru360 but decreased single-channel activity in UCP2^-/-^ mitoplasts. Finally, our detection of a reduced Ru360 sensitive (Ca^2+^)m in intact cardiomyocytes from UCP2^-/-^ mice further confirmed this observation. Since none of our experiments neither showed a complete inhibition of mitochondrial Ca^2+^ uptake in isolated mitochondria, a fully inhibited mCa1 activity in the single channel experiments nor a completely reduced (Ca^2+^)m under basal conditions, we conclude that UCP2 is not essential for mitochondrial Ca^2+^ uptake as previously suspected [23]. This conclusion is consistent with recent reports that were able to characterize the mitochondrial Ca^2+^ uniporter as a highly Ca^2+^-selective protein complex that consists of the pore-forming mitochondrial Ca^2+^ uniporter protein (MCU) [50, 51], the essential MCU regulator (EMRE), and the mitochondrial calcium uptake 1 and 2 (MICU1/2) [52–55], which regulate mitochondrial Ca^2+^ handling. Our data suggests that in the heart, through interaction with mCa1, UCP2 could operate cooperatively or sequentially for the modulation of transporting Ca^2+^ across the inner mitochondrial membrane as another part of this complex system. However, regulatory effects on additional mitochondrial Ca^2+^ channels or Ca^2+^ uptake mechanisms in the heart should also be considered. Of note, in a recent study in HeLa cells UCP2 was reported to regulate the extra-large mitochondria calcium channel (*xl*-MCC), that essentially contains MCU and EMRE. Notably, UCP2 was shown to have a much higher affinity to this particular channel than to other MCU-dependent calcium currents (i.e. the intermediate mitochondria calcium channel *i*-MCC) [56].

Previous studies also described an inhibitory effect of adenine nucleotides especially ATP on mCa^2+^-uptake [16] and on UCP2 function, respectively [17, 18, 22]. Our data from WT mice collected 1.) by patch-clamping the inner mitochondrial membrane and 2.) by testing dual Ca^2+^ transients from the mitochondrial ((Ca^2+^)m) and intracellular compartment ((Ca^2+^)c) in whole-cell patch-clamped, isolated, intact cardiomyocytes confirmed MCU inhibition by ATP. However, no ATP effect was observed in UCP2^-/-^ mice, supporting the notion that the inhibition of mCa^2+^-uptake by ATP is mediated via UCP2. Since our whole-cell experiments revealed decreased (Ca^2+^)m using high concentrations of the nucleotide (15 mM) and increased (Ca^2+^)m in the absence of ATP as compared to physiological ATP concentrations (5 mM) we suggest that under physiological conditions MCU activity is diversely dependent on the intracellular ATP concentration. In addition, previous data reported the MCU to be involved in the regulation of mitochondrial energy (ATP) production [1, 2]. Thus, under physiological conditions UCP2 seems to serve as a regulator of the respiratory chain via the modulation of mCa^2+^-uptake indicating a physiological feedback mechanism in situations with high energy demands.

An alternative explanation as to how UCP´s and ATP might regulate mitochondrial Ca^2+^ uptake could be the modulation of (Ca^2+^)m by increased ATP and increased SERCA activity leading to reduced depletion of the internal Ca^2+^ stores as observed by De Marchi et al. in intact HeLa cells under UCP3 depletion [57]. In our study though SR Ca^2+^ load was not different between WT vs. UCP2^-/-^ we did not specifically monitor SERCA activity. This needs to be further investigated in future studies.

The inhibitory effect on (Ca^2+^)m and subsequent upregulation of (Ca^2+^)c by Ru360 at concentration of 10 nM, as well as the inhibitory effect on both (Ca^2+^)m and (Ca^2+^)c in the presence of 100 nM Ru360 in WT cardiomyocytes were consistent with a previous report [9]. A possible explanation for the upregulation of (Ca^2+^)c in the presence of 10 nM Ru360 is that moderate increases in ROS production activate RyRs and therefore (Ca^2+^)c. This suggest a close interplay of mitochondrial and cytosolic redox state/ROS production and elementary Ca^2+^ release events as previously described. We found no evidence for an unspecific Ru360 effect at 10 nM, e.g. by downregulation of cytosolic Ca^2+^ release which subsequently affects mitochondrial Ca^2+^ uptake, Additional control experiments analyzing cytosolic Ca^2+^ uptake using only Fluo4-K5 in the presence or absence of 10 nM Ru360 also confirmed this observation (data not shown).

We provide an additional explanation for the observations at 10 nM Ru360 since a shift of available dyadic cleft Ca^2+^ in WT cardiomyocytes caused by inhibited mCa^2+^-uptake could lead to an increased (Ca^2+^)c transient, possibly due to the spatial vicinity of these structures in an area of limited diffusion like the dyadic cleft. This hypothesis is further being supported by our data using an alternative mode of inhibiting mCa1/MCU, i.e. elevation of (Ca^2+^)c with high ATP concentrations in isolated cardiomyocytes, which should not influence RyRs in WT mice but also inhibited (Ca^2+^)m.

Furthermore, our finding that the absence of ATP in WT cardiomyocytes led to an increase of (Ca^2+^)m and subsequent downregulation of (Ca^2+^)c supports the notion of modulated local Ca^2+^ distribution based upon the ATP dependent modulation of mCa1/MCU as mentioned above.

Higher Ru360 concentrations are known to inhibit the RyR, which could explain the diminished (Ca^2+^)c in the presence of 100 nM Ru360 [58] in WT and UCP2^-/-^. Strikingly, the amplitude of (Ca^2+^)c in UCP2^-/-^ mice was not significantly altered vs. WT (p>0.05) while (Ca^2+^)m was reduced. Since cytosolic Ca^2+^ overload is considered as unfavorable and is observed in the onset of heart failure [59], compensatory mechanisms in UCP2^-/-^ mice are likely. We hypothesize that the decreased transsarcolemmal Ca^2+^-influx in UCP2^-/-^ vs. WT is such a mechanism. A similar modulation where reduced Ca^2+^ influx was observed as a potential compensatory mechanism against Ca^2+^ overload has been shown in NCX knockout mice [44].

With regard to our experiments analyzing transsarcolemmal Ca^2+^ influx it should be noted, that De Marchi et al. showed that Thapsigargin can also directly reduce (Ca^2+^)m in HeLA cells [57]. A subsequent paper revealed that under thapsigargin, mitochondrial Ca^2+^-uptake apparently uses alternative, Letm1-dependent routes in the same cell model [60]. This mechanism could also explain our observations but it does not clarify why a diminished transsarcolemmal Ca^2+^-influx should occur in UCP2^-/-^ cardiomyocytes only while using identical amounts of thapsigargin in both groups. Additionally, we analyzed murine cardiomyocytes and it is unclear whether the observations by De Marchi et al. and Waldeck-Weiermair et al. in HeLA cells can be transferred to our results. This should be addressed in future studies with focus on SERCA-activity and (Ca^2+^)m in intact murine cardiomyocytes.

Since we observed a trend towards a diminished peak cytosolic Ca^2+^ transient in UCP2^-/-^ vs. WT, a reduction of mitochondrial Ca^2+^-transients in UCP2^-/-^ vs. WT is possibly directly attributable to this observation. This suggests either a diminished SR Ca^2+^-load, altered RyR kinetics or differences in transsarcolemmal Ca^2+^ influx in UCP2^-/-^ vs. WT as alternative explanations.

However, we found no difference in SR Ca^2+^ load but detected a significant alteration in (Ca^2+^)c rate of rise suggesting modulated RyR kinetics in UCP2^-/-^ vs. WT. We did not further analyze this aspect, which limits the conclusions in our paper. It should also be further investigated in future studies.

In summary our study supports an essential modulating role for UCP2 in mCa^2+^-uptake, which most likely has a direct impact on Ca^2+^ handling of the entire murine cardiomyocyte under steady state conditions and is modulated by ATP. The quantities of mitochondrial Ca^2+^ uptake on a beat-to-beat basis, however, remain an area of debate, and should be rather low according to recent data by other groups [61, 62].

Our data also indicate that UCP2 inhibition by ATP could serve as a potential regulatory mechanism in controlling energy production under physiological conditions via the MCU. Compensational mechanisms seem to protect UCP2^-/-^ mice from these otherwise disadvantageous Ca^2+^ regulations.
